# The 2.4 Å cryo-EM structure of a heptameric light-harvesting 2 complex reveals two carotenoid energy transfer pathways

**DOI:** 10.1126/sciadv.abe4650

**Published:** 2021-02-12

**Authors:** Alastair T. Gardiner, Katerina Naydenova, Pablo Castro-Hartmann, Tu C. Nguyen-Phan, Christopher J. Russo, Kasim Sader, C. Neil Hunter, Richard J. Cogdell, Pu Qian

**Affiliations:** 1 Institute of Molecular, Cell and Systems Biology, Glasgow University, Glasgow G12 8QQ, UK.; 2 MRC Laboratory of Molecular Biology, Francis Crick Avenue, Cambridge CB2 0QH, UK.; 3 Materials and Structural Analysis, Thermo Fisher Scientific, Achtseweg Noord 5, 5651 GG Eindhoven, Netherlands.; 4 Department of Molecular Biology, The University of Sheffield, Sheffield S10 2TN, UK.

## Abstract

We report the 2.4 Ångström resolution structure of the light-harvesting 2 (LH2) complex from *Marichromatium* (*Mch.*) *purpuratum* determined by cryogenic electron microscopy. The structure contains a heptameric ring that is unique among all known LH2 structures, explaining the unusual spectroscopic properties of this bacterial antenna complex. We identify two sets of distinct carotenoids in the structure and describe a network of energy transfer pathways from the carotenoids to bacteriochlorophyll *a* molecules. The geometry imposed by the heptameric ring controls the resonant coupling of the long-wavelength energy absorption band. Together, these details reveal key aspects of the assembly and oligomeric form of purple bacterial LH2 complexes that were previously inaccessible by any technique.

## Teaser

The structure of a heptameric LH2 antenna complex reveals energy transfer pathways and basic assembly principles.

## INTRODUCTION

In purple bacterial photosynthesis, light energy absorbed by light-harvesting 2 (LH2) complex is transferred via the LH1 complex to the reaction center complex, where it is trapped by the primary charge separation reactions. A detailed understanding of the molecular mechanisms of these reactions is firmly underpinned by knowledge gained from the structures of reaction center and light-harvesting complexes (*1*–*8*). In this regard, the structures of the membrane-bound pigment-protein complexes from purple photosynthetic bacteria have been particularly influential. The most abundant of these is the LH2 antenna complex. All such complexes are formed by the circular oligomerization of dimer building blocks consisting of pairs of low–molecular weight, hydrophobic apoproteins to which bacteriochlorophyll (BChl) and carotenoid (Car) molecules are noncovalently bound. The principles underlying the assembly, oligomerization, ring size, and absorption properties of many natural variants of LH2 complexes are required for future designs of genetically modified and synthetic light absorbers, and therefore, structural details are needed. Yet, x-ray crystallographic studies have generally encountered difficulties with weak protein-protein contacts, lattice disorder, and poor diffraction. High-resolution single-particle cryogenic electron microscopy (cryo-EM) structures, required to correlate fine structural details with spectroscopic analyses, have, until now, been hampered by the relatively small (<120 kDa) sizes of LH2 complexes and the variable amounts of radiation damage incorporated into the final reconstructed maps. We used a recently developed specimen support, which allows imaging without specimen movement and radiation damage artefacts (*9*), to determine the 2.4 Å resolution structure of the LH2 complex from the marine purple bacterium *Marichromatium* (*Mch.*) *purpuratum*. We show how the circular packing of seven α/β subunits creates binding sites for two populations of carotenoid pigments, and how this organization determines the near-infrared (NIR) absorption of excitonically coupled BChl *a* molecules.

## RESULTS

**Fig. 1:**
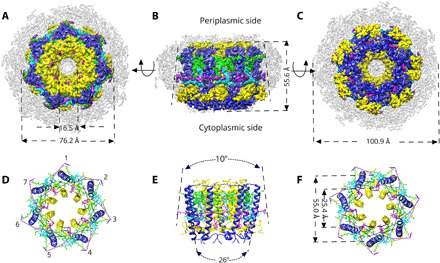
Cryo-EM structure of the LH2 from *Mch. purpuratum*. (**A** to **C**) The cryo-EM map of the LH2 complex is displayed according to the color code below, and views from the cytoplasmic side of the membrane (A), in the membrane plane (B), and from the periplasmic side of the membrane (C) are shown. (**D** to **F**) Ribbon models corresponding to the views and coloring in (A) to (C). The α/β heterodimers are numbered in (D). (E) View of the complex in the membrane plane as in (B), showing the marked tapering of trans-membrane helices and the reversed 10^o^ taper and trapezoidal shape of the LH2 complex. The diameters of the inner α and outer β rings of the LH2 in (F) are measured from the centers of the respective helices, midway through the transmembrane region of the complex. Color code: α-polypeptide, yellow; β-polypeptide, medium blue; B828, green; B800, cyan; Car1, red; Car2, purple; detergent belt, gray.

Briefly, 17,338 electron cryomicrograph movies were collected, and 3.05 million particles were picked for data processing. Figure 1 (A to C) shows three views of the cryo-EM map of the *Mch. purpuratum* LH2 complex at 2.4 Å resolution, which is a previously unidentified, circular LH2 structure formed of seven α/β apoprotein heterodimer subunits. Seven α-apoprotein α helices form an inner ring of 25.4 Å diameter and the seven β-apoprotein α helices form an outer ring of 55.0 Å diameter. The complex is surrounded by a 10-Å-wide belt of disordered detergent molecules, and the central hole has a diameter of 16 Å. Viewed from the side, at right angles to the long axes of the helices, the profile of the complex is notably trapezoidal, being broader on the cytoplasmic side. This feature is enhanced compared with the previous x-ray structures of LH2 complexes (*3*, *4*, *10*).

Previous mass spectrometry analyses (*11*) found three homologous α-polypeptides and three homologous β-polypeptides in the purified *Mch. purpuratum* LH2 complex (figs. S1 and S2). The exact distribution of the different but highly homologous α/β-pairs in a single LH2 molecule is unknown. A 2.8 Å resolution reconstruction of the LH2 without symmetry imposed (fig. S3) shows a pseudo-C7 symmetry up to this resolution (fig. S4B). This indicates that either the different α/β-pairs are randomly distributed in the heptameric LH2 ring and/or the different α/β-pairs have very similar structures. Comparison of the amino acid sequences of the three different α-polypeptides (fig. S1) shows that they differ in their N termini (residues 1 to 15) and at residue α-47 (M or L), which lies in the helical region. The relatively low resolution and high atomic *B*-factors in the N-terminal region of the α-polypeptide in the structure (fig. S4D) are probably due to mixed densities from the three different sequences and the intrinsic flexibility of the loop, which is also seen in the C-terminal region. Only two residues differ in the central helical regions of the three types of β-polypeptide—positions β-14 (A or E) and β-43 (V or I)—and the N- and C-terminal loops are much shorter than for the α-polypeptides. Because none of these differences could be resolved, we imposed C7 symmetry on the three-dimensional (3D) reconstruction, producing an LH2 structure close to the native LH2 from *Mch. purpuratum.* The cryo-EM densities for the four nonconserved residues α-47 (M or L), β-12 (T or S), β-14 (A or E), and β-43 (V or I) are shown in fig. S5, with the alternative side chains modeled.

**Fig. 2:**
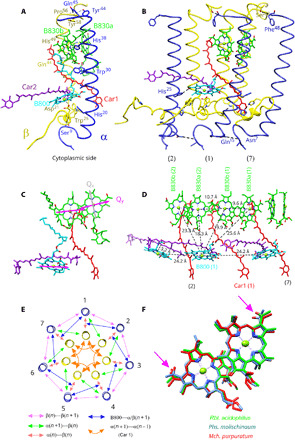
Protein-protein and protein-pigment interactions in the LH2 from *Mch. purpuratum*. (**A**) The molecular model of one subunit of the LH2 complex is shown. Only the residues involved in protein-protein and protein-pigment interactions are labeled for clarity. (**B**) Three subunits are presented to show the interactions between them, but for clarity, only one unit includes pigments. The phytol tails of the BChls are omitted. (**C**) All the pigments within one subunit are shown; the BChl *a* Q_x_ and Q_y_ transitions are indicated in gray and magenta, respectively. (**D**) The pigment arrangement in three consecutive subunits of the LH2 complex, showing the distances between Mg atoms of the BChls. The BChl *a* phytol tails are omitted. (**E**) All protein-protein and protein-pigment interactions in the LH2 complex are summarized schematically. (**F**) The B828 pair from *Mch. purpuratum* (red), the B858 pair from *Rbl. acidophilus* (green), and *Phs. molischinaum* (cornflower blue) are superimposed for comparison. The positions of the C-13^2^ carbonyl group of BChl *a* are indicated by pink arrows. The color code is the same as in Fig. 1: α-polypeptide, yellow; β-polypeptide, medium blue; B828, green; B800, cyan; Car1, red; Car2, purple.

The complete α-apoprotein can be traced in the structure apart from the N- and C-terminal amino acids, and the β-apoprotein can be fully traced apart from the first four N-terminal amino acids. Both apoproteins have a single membrane-spanning α helix, with their N termini on the cytoplasmic side of the complex and their C termini on the periplasmic side (Fig. 2A). There are two strong H-bonds between adjacent heterodimers, between α-Ser^63^ and β-Phe^48^ on the periplasmic side, and between β-Gln^15^ and β-Asn^7^ on the cytoplasmic side (Fig. 2B). Each α/β heterodimer binds, noncovalently, three molecules of BChl *a* and two molecules of the carotenoid, okenone. Two of the BChl *a* molecules form a strongly interacting dimer with their Mg^2+^ atoms liganded by two His residues, α-His^49^ and β-His^38^. The distance between these two Mg^2+^ atoms is 9.6 Å. By analogy with previous LH2 structures, the 828 nm absorption band is assigned to these two BChl *a* molecules. Their orientations are fixed by a set of H-bonds between β-Trp^30^ and the C-17^3^ carbonyl group of the α–BChl *a* molecules and between the C-13^2^ carbonyl group of the β-BChls *a* and α-Gln^44^. The third monomeric BChl *a* molecule is located on the cytoplasmic side of the complex; its Mg^2+^ atom is liganded to α-Asp^21^, and there is an H-bond between its C-3^1^ carbonyl group and β-His^25^ from the β-apoprotein in the next heterodimer (Fig. 2B). This BChl *a* molecule can be assigned to the 800 nm absorption band. One molecule of the carotenoid okenone (Car1) has a twisted, all-trans configuration (fig. S6) and runs approximately parallel to the heterodimer α helices across most of the length of the complex. The region of its conjugated C═C double bonds comes into close contact (3.6 Å) with the bacteriochlorin ring of the B828 α–BChl *a* molecule and runs parallel to the Q_y_ transition dipole moment of that bacteriochlorin ring (Fig. 2C). A second okenone molecule (Car2) adopts a 9-cis configuration (fig. S6) and lies in the plane of the membrane, at right angles to the heterodimer α helices. Its region of conjugated C═C double bonds run parallel to the Q_x_ transition dipole moment of the B800 BChl *a* at a closest distance of 3.2 Å (Fig. 2, C and D). The presence of this Car2 in every heterodimer subunit and the fact that it adopts a 9-cis configuration are both unique features of this LH2 complex. All inter-/intra-subunit protein-protein and protein-pigment interactions are summarized in Fig. 2E.

## DISCUSSION

Three basic factors determine the position of the long-wavelength Q_y_ absorption band arising from the ring of tightly coupled BChl *a* molecules in purple bacterial LH2 complexes (*7*, *12*, *13*): (i) the “site” energy of the individual BChl *a* molecules in their respective binding sites, (ii) the coupling between BChl *a* molecules within their α/β subunits to form a dimer, and (iii) the interactions between dimers in adjacent α/β subunits around the LH2 ring. All these parameters of the LH2 complexes from *Mch. purpuratum*, *Phaeospirillum* (*Phs.*) *molischianum*, and *Rhodoblastus* (*Rbl.*) *acidophilus* are summarized in table S2 for comparison. In the case of LH2 from *Rbl. acidophilus* [formerly *Rhodopseudomonas* (*Rps.*) *acidophila*] the strongly coupled ring of BChl *a* molecules has a Q_y_ absorption band at 858 nm, whereas in the LH2 complex from *Phs. molischianum* and *Mch. purpuratum*, this absorption band is blue shifted to 846 nm (*14*) and 828 nm, respectively (fig. S7). The organization, orientation, and H-bonding of the bacteriochlorin rings of both BChl *a* dimers are very similar among these three species (Fig. 2E), in terms of in-plane orientation of their carbonyl groups relative to the plane of the bacteriochlorin rings, and their Mg^2+^-Mg^2+^ distances, which are 9.6 Å in the case of *Mch. purpuratum* and 9.4 Å in the case of *Phs. molischianum* and *Rbl. acidophilus*. Although these similarities could suggest similar dimer exciton bands in these LH2 complexes, there is a large difference in the Mg^2+^-Mg^2+^ distances between one of the BChl *a* molecules in a heterodimer subunit and the nearest BChl *a* molecule in the next heterodimer subunit in the LH2 ring. In the case of *Rbl. acidophilus* and *Phs. molischinaum*, this distance is 8.8 and 8.9 Å, whereas for *Mch. purpuratum*, it is 10.7 Å (Fig. 2D). This increased separation, as well as the increased angle between dimers imposed by the 7-mer ring relative to a 9-mer, lowers the strength of dimer-dimer exciton coupling, which causes the blueshift of the B858 band in *Rbl. acidophilus* to B828 in *Mch. purpuratum*.

**Fig. 3:**
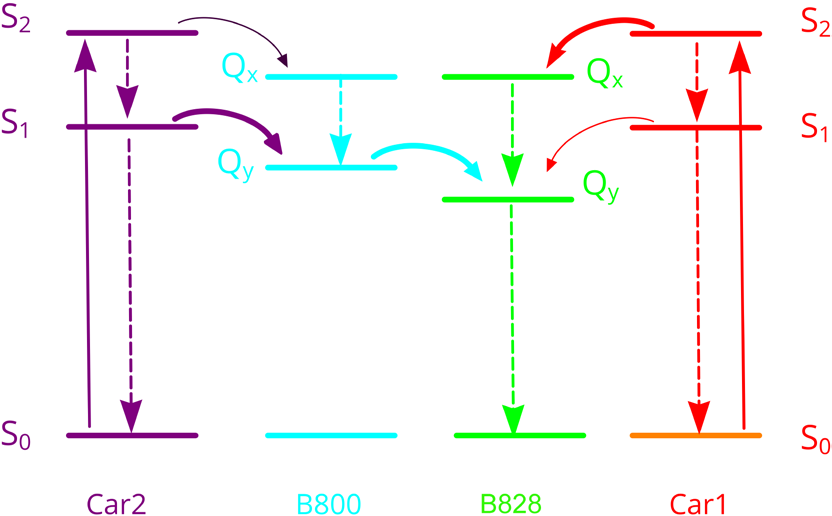
Schematic diagram of energy levels and energy transfer pathways from Car to BChl *a* in the LH2 complex from *Mch. purpuratum.* Solid curved arrows represent energy transfer pathways, and dashed arrow lines denote internal conversions. Color code is same as in Fig. 1. Less important, possible energy transfer pathways from Car1 to B800 and Car2 to B828 are omitted for brevity.

Carotenoids have a strong visible absorption band arising from the optically allowed transition from the ground state, S_0_, to the second excited singlet state, S_2_ (Fig. 3) (*15*, *16*). The lower lying, symmetry-forbidden first excited singlet state, S_1_, is populated by ultrafast internal conversion from the S_2_ state (Fig. 3) (*17*, *18*). In most purple photosynthetic bacteria, carotenoids function as accessory light-harvesting pigments and can transfer absorbed solar energy to the BChl molecules, thereby making it available to drive photosynthesis (*19*). A previous study of ultrafast energy transfer within isolated LH2 complexes from *Mch. purpuratum* showed that the efficiency of energy transfer from okenone to BChl *a* is 95 ± 5% (*20*). This Car–to–BChl *a* energy transfer can take place either from the Car S_2_ state alone or from both the S_2_ and S_1_ states, depending on the type of carotenoid involved. One okenone transfers its excitation energy to B828 in <200 fs from S_2_ and in 3.8 ps from S_1_, and the other transfers its excitation energy to B800 in about 0.5 ps (Fig. 3). The results of this study could only be explained by assuming the presence of two separate pools of okenone with different orientations of their transition dipole moments relative to BChl *a* molecules. The structure of the LH2 complex from *Mch. purpuratum* described here is the first direct observation of these two populations of different carotenoids, which serve structurally and spectroscopically distinct BChl *a* molecules, and it accounts for the spectroscopic properties of these pigments, which have lain unexplained for more than 24 years. The 3.6 Å distance (Fig. 2B) is consistent with the rapid (<200 fs) energy transfer from Car1 to the nearest B828 pigment, and Car2 is precisely positioned, with a distance of 3.2 Å, to transfer its excitation energy to B800. The pigment organization and their configurations in the LH2 complex are shown in figs. S8 and S9.

**Fig. 4:**
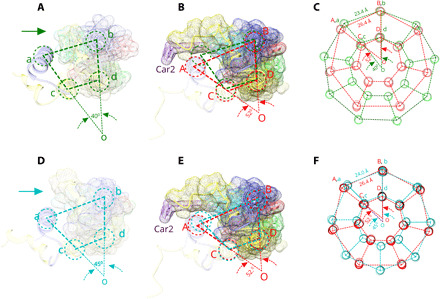
Assembly mechanism of LH2 complexes from *Rbl. acidophilus*, *Mch. purpuratum*, and *Phs. molischinum.* An LH2 subunit pair from *Rbl. acidophilus* (**A**), *Mch. purpuratum* (**B**), and *Phs. molischinum* (**D**), viewed from the cytoplasmic side of the membrane. An arrow points to N-terminal domain of the α-polypeptide of *Rbl. acidophilus* in (A) and *Phs. molischinum* in (E). The dashed green/red/light sea blue circles represent the positions of trans-membrane helices of the LH2 subunits. The wider A-B spacing (red) is compatible with the second okenone molecule, Car2 (purple), and a longer N-terminal region of the α-polypeptide (yellow), whereas the shorter a-b (green and blue) spacing is not (B and **E**). (**C** and **F**) The schematic representation of LH2 complexes from *Rbl. acidophilus* (green), *Mch. purpuratum* (red), and *Phs. molischinum* (light sea blue), with their segments in as in (A), (B), and (D) illustrates their three different patterns of oligomerization.

Trying to understand the factors that control the ring size of purple bacterial LH2 complexes is a long-standing problem. Figure 4 compares the details of the interactions of the α/β heterodimers in the structures of the LH2 complexes from *Rbl. acidophilus* (9-mer), *Phs. molischinum* (8-mer), and *Mch. purpuratum* (7-mer). This comparison provides a clear explanation for their different oligomeric sizes.

In the case of the 9-mer LH2 complex from *Rbl. acidophilus*, a line drawn from the center of a pair of β helices (ab), followed by a pair of lines from the center of each β helix to the center of their heterodimer α apoprotein helix (ac and bd), forms a segment subtending a 40° angle (∠aob = 360°/9 = 40°) (Fig. 4A). In the case of the 7-mer LH2 complex from *Mch. purpuratum*, each α/β heterodimer relates to the adjacent segment by a ~52° rotation (∠AOB = 360°/7 = 52.14°) (Fig. 4B). This corresponding angle in the LH2 from *Phs. molischianum* changes to 45° (∠aob = 360°/8 = 45°) (Fig. 4D). Figure 4C illustrates how 9- and 7-mer patterns of oligomerization are manifested at the level of intact LH2 complexes. This comparison provides new insights into ways that LH2 α/β heterodimers fit together and particularly the basis for the assembly of a 7-mer ring in the *Mch. purpuratum* LH2 antenna complex. First, as shown in Fig. 4B, the longer N-terminal region of the α apoprotein and the second okenone molecule, Car2, protrude from the α/β heterodimer in a way that would induce strong steric clashes if the segment angle was reduced to 40° as in the case of 9-mer *Rbl. acidophilus*. A wider segment angle, namely ~52°, is needed to circumvent these potential clashes necessitating a 7-mer complex. The same method was used for comparison between 8-mer LH2 from *Phs. molischinaum* and 7-mer LH2 from *Mch. purpuratum*. Figure 4D shows the assembly pattern of the 8-mer LH2. If this pattern is applied for the *Mch. purpuratum* LH2, the position of next β helix is still too close to Car2 and the N-terminal domain of the α apoprotein (Fig. 4E). Their oligomerizations are illustrated in Fig. 4F. Therefore, as a direct consequence of this work, we can clearly visualize the main factors involved in controlling this segment angle and, thereby, the oligomeric size of the LH2 ring, namely the existence of the second carotenoid and longer N-terminal domain of the α apoprotein. The LH2 complexes from *Phs. molischianum* and *Rbl. acidophilus* have no second carotenoid. The length of the N-terminal domain of the α apoprotein plays a key role in oligomerization. The α apoprotein of *Phs. molischianum* is three amino acids longer than that of *Rbl. acidophilus* (fig. S1). These three extra amino acids produce a bigger volume (fig. S10), which affects the spacing of β apoprotein in the LH2 complex, resulting in an 8-mer LH2. The LH2 from *Rhodobacter sphaeroides* is a 9-mer (*21*). Its N-terminal domain of the α apoprotein has the same length as that in *Rbl. acidophilus* (fig. S1).

The extended N-terminal domain for LH2α modifies the packing of α/β heterodimers, widening their spacing by 12° and creating space for binding an extra carotenoid (Fig. 4B). This packing of neighboring α/β-apoprotein dimers in the holo-LH2 structure also determines the overall oligomeric ring size, the number of excitonically coupled BChl *a* molecules, the extent of coupling, and the position of the long-wavelength absorption band. Thus, the structure of the 7-mer LH2 complex from *Mch. purpuratum* provides a detailed structural understanding of how the oligomeric ring size of LH2 complexes is controlled and explains its previously determined spectroscopic and biochemical properties.

Many species of purple bacteria have multiple *puc* genes encoding LH2 apoproteins, suggesting that the heterogeneous LH2 complexes, made from different α/β-apoprotein pairs, are much more common than previously appreciated (*22*, *23*). Our structural analysis of such a heterogeneous complex, from *Mch. purpuratum*, by cryo-EM circumvents the difficulties encountered over many years in x-ray crystallography, where heterogeneous LH2 complexes weaken protein-protein contacts, leading to long-range internal disorder within the lattice and poor diffraction. Technical developments in cryo-EM, including direct electron detectors, high-speed data acquisition, movement-free specimen supports, and image processing algorithms circumventing radiation damage, have enabled us to determine the structure of this small, heterogeneous LH2 complex to a resolution sufficient to identify and measure the critical features of the pigments and understand the energy transfer pathway.

## MATERIALS AND METHODS

### Protein purification

*Mch. purpuratum* strain BN5500 (also designated as DSM1591 or 984) was grown anaerobically in the light in Pfennig’s medium (*24*), with incandescent bulbs at a light intensity of ~80 μmol m^−2^ s^−1^ at 30°C. Harvested cells were washed once with 20 mM MES and 100 mM KCl (pH 6.8), and the pellet was flash-frozen until required. The cell pellet was defrosted and resuspended in 20 mM Tris-HCl buffer (pH 8.0), with a few grains of deoxyribonuclease and MgCl_2_ added, homogenized, and then disrupted by passing twice through a French press cell at ~15,000 psi. Unbroken cells and debris were removed by a low-speed spin (3000*g*, 10 min, 4°C) and the chromatophore membranes were pelleted by ultracentrifugation (180,000*g*, 90 min, 4°C). The chromatophores were resuspended in 20 mM Tris-HCl (pH 8.0) buffer to an optical density = 50 cm^−1^ at the NIR absorbance maximum (~828 nm). The sample was solubilized by the addition of *N*,*N*-dimethyldodecylamine *N*-oxide to 1.0% (v/v), with stirring for 1 min, and the mixture was immediately loaded on to a preequilibrated glass gravity Q Sepharose (GE Healthcare) column. The sample was washed with copious amounts of 0.02% *n*-dodecyl-β-D-maltopyranoside (DDM) in 20 mM Tris-HCl (pH 8.0) (called TD buffer), and then the LH2 was eluted with increasing concentrations of NaCl in TD buffer. The LH2-rich fractions were assayed, pooled, and dialyzed overnight in TD buffer to remove the NaCl. Because of the huge amount of free pigment involved, the process was repeated the following day by loading the dialyzed sample on to a fresh Q Sepharose column. The eluted LH2 was then assayed, pooled, and concentrated before passage down a Superdex G200 gel filtration column (GE Healthcare) with TD buffer. Fractions having an *A*_828_/*A*_280_ ratio of 2.2 or higher were concentrated to an absorbance at 828 nm = 100 cm^−1^ for cryo-EM grid preparation.

### Cryo-EM data collection

Two different grids were used for cryo-EM specimen preparation. Initially, a QuantiFoil R1.2/1.3 400-mesh Cu grid was glow-discharged for 60 s (easiGlow). The grid was plunge-frozen into liquid ethane using a FEI Vitrobot MK IV, equilibrated to 100% humidity at 4°C. A sample volume of 3 μl was applied to the grid, which was blotted for 2.5 s before freezing. For the final data collection, a HexAufoil grid (*9*), manufactured in-house at the Laboratory of Molecular Biology (LMB), was used. The grid was plasma-cleaned under a mixed atmosphere (O_2_:Ar = 1:9) in a plasma chamber for 60 s (Fischione 1070) and then vitrified using a manual plunger of the Talmon type (*25*) in a 4°C cold room, and an ethane cryostat (*26*) held at 93 K. Three microliters of protein solution were applied to the foil side of the cleaned grid and manually blotted for 11 s with filter paper (Whatman #1). All grids were stored in liquid nitrogen until use. Data were collected on a Thermo Fisher Titan Krios G3i cryogenic electron microscope equipped with a Falcon 4 direct electron detector at the Cambridge Pharmaceutical Cryo-EM Consortium (*27*). The microscope was operated at 300 kV with a nominal magnification of 120,000×, corresponding to 0.646 Å/pixel at the specimen level, calibrated using the Au (111) lattice reflections of the foil. The detector was operated in counting mode at a flux of 3.58 e^−^/Å^2^ per second. Each 12.18 s exposure was fractionated into 42 frames, resulting in an electron fluence of 1.04 e^−^/Å^2^ per frame. The defocus range was set to −0.8 to −2.4 μm. Automated data acquisition was performed in EPU 2.6 (Thermo Fisher Scientific) with one exposure per hole in aberration-free image shift mode. In total, 7795 movies were collected from the QuantiFoil R1.2/1.3 grid, and 8935 movies were collected from the HexAuFoil grid (fig. S11).

### Cryo-EM data processing

The initial dataset collected from the QuantiFoil grid was processed in RELION 3.1. The movie stacks were motion-corrected within RELION (*28*) on 5 × 5 patches. The contrast transfer function (CTF) parameters were determined using Gctf (*29*). The particles were auto picked in cisTEM (*30*), and their coordinates were imported into RELION for particle extraction using a box size of 270 × 270 pixels. A total of 1,723,876 particles were extracted and subjected to 2D reference-free classification, and then 1,337,902 particles were selected from good 2D classes. Reference-free 2D classification showed that the LH2 from *Mch. purpuratum* is a heptamer, which, in terms of its overall circular arrangement of subunits, has an architecture similar to other LH2 complexes. The initial heptamer model for 3D classification was built from an α/β subunit taken from the LH2 of *Phs. molischianum* [Protein Data Bank (PDB): 1LGH] using Chimera (*30*). At this stage, C7 symmetry was imposed for 3D reconstructions. The best 3D class (3.98 Å), of four classes, contained 867,046 (50.3%) particles. After multiple rounds of 3D refinement, anisotropic magnification, beam tilt, trefoil, fourth-order aberration, per-particle defocus, and per-micrograph astigmatism estimation, and particle movement tracking using Bayesian polishing with the default parameters in RELION, these 867,046 particles produced a 2.48 Å resolution map.

The dataset from the HexAuFoil grid was processed according to the previously proposed method for handling movement-free cryo-EM data (*28*). The whole micrograph movement was corrected using MotionCorr (*31*). The motion-corrected stacks were imported into Relion 3.1. A total of 1,330,145 particles was auto picked in cisTEM. The CTF correction was performed in CTFFIND4.1 (*32*). The particles were extracted into 270 × 270 pixel boxes and subjected to 3D classification with a reference taken from previously determined map with a 30 Å initial low-pass filter applied. In total, 414,511 (31.2%) particles from the best 3D class (of four classes) were selected. The selected particles were reextracted using 512 × 512 box size for CTF refinement and Bayesian polishing (*33*). No significant particle movement could be fit by the Bayesian polishing, as judged by the resolution of the reconstruction before and after this step. Similarly, no significant aberrations or anisotropic magnification were found. Per-particle defocus and/or astigmatism refinement did not yield any improvement compared to per-micrograph estimation of these parameters. We also tried separating the data into 149 optical groups based on image beam shift values and estimating the optical aberrations for each group separately, but no significant variation between these groups was found. The final 3D reconstruction reached 2.38 Å resolution with C7 symmetry (2.76 Å without symmetry). Per-frame reconstructions were produced from particles extracted at their refined positions from individual frames of the aligned stacks. These reconstructions were used to extrapolate the structure factors, i.e., phases and amplitudes, in the 10 to 2.5 Å resolution range to zero dose, yielding the final map (*9*).

### Modeling and refinement

A α/β subunit polypeptide pair taken from the LH2 complex of *Phs. molischianum* (PDB: 1LGH) was docked into the C7 symmetry imposed cryo-EM map as a template using Chimera (*34*) such that three BChl *a* molecules were roughly fitted with their corresponding densities. Matrix-assisted laser desorption/ionization–time-of-flight mass spectroscopy of the *Mcr. purpuratum* LH2 revealed that three different α- and three different β-polypeptides are incorporated into the LH2 complex of *Mch. purpuratum*. They are distributed randomly, and with unknown stoichiometries, in the LH2 complex. Imposing C7 symmetry on the cryo-EM map of the LH2 from *Mch. purpuratum* complex during refinement mixed three different α- or β-polypeptides together, resulting in an averaged single α- and single β-polypeptide in the map of the LH2 complex. In this case, the longest α– and β–amino acid sequences, i.e., α3 and β2 (fig. S1) were selected for mutation of amino acids in the template using COOT (*35*). The carotenoid lycopene in the template was replaced with an all-trans okenone. The second carotenoid was fitted with confidence by 9-cis okenone (figs. S6 and S9). Thus, a subunit of the LH2 from *Mch. purpuratum*, α_1_β_1_Car_2_BChl*a*_3_, was constructed. This subunit was then copied into the LH2 map using the rigid body fitting in Chimera, forming an atomic model of the heptameric LH2 complex. The model was real space refined in COOT (*35*). A geometry-optimized model was then subjected to global refinement using REFMAC5 (*36*) and Phenix (*37*). The refinement statistics are summarized in table S1. The refined model and its cryo-EM map were deposited in the PDB and the Electron Microscopy Data Bank (EMDB) with codes of 6ZXA and EMD-11516.

## SUPPLEMENTARY MATERIALS

Supplementary material for this article is available at http://advances.sciencemag.org/cgi/content/full/7/7/eabe4650/DC1

## Appendix

View/request a protocol for this paper from *Bio-protocol*.
